# Eliminating prediction bias in CO_2_ emission models for lactating cows by incorporating feed intake: accurate quantification of methane-reducing effects using a CO_2_-based method, demonstrated by a case study on 3-nitrooxypropanol

**DOI:** 10.5713/ab.250867

**Published:** 2026-03-11

**Authors:** Kohei Oikawa, Fuminori Terada, Mitsunori Kurihara, Tomoyuki Suzuki, Itoko Nonaka, Kenji Hosoda, Yuko Kamiya, Sanggun Roh, Satoshi Haga

**Affiliations:** 1 Institute of Livestock and Grassland Science, National Agriculture and Food Research Organization (NARO), Ibaraki, Japan; 2 Graduate School of Agricultural Science, Tohoku University, Miyagi, Japan; 3 Mito Research Center, Meiji Feed Co., Ibaraki, Japan; 4 Office of Smart Sustainable Agri-food Systems, National Agriculture and Food Research Organization (NARO), Ibaraki, Japan; 5 Institute of Livestock and Grassland Science, National Agriculture and Food Research Organization (NARO), Tochigi, Japan; 6 Kyusyu-Okinawa Agricultural Research Center, National Agriculture and Food Research Organization (NARO), Kumamoto, Japan

**Keywords:** Dry Matter Intake, Holstein, Methane Emissions, Sniffer Method

## Abstract

**Objective:**

The methane (CH_4_) emission prediction method, using predicted CO_2_ emissions and the CH_4_:CO_2_ concentration ratio, faces challenges in evaluating the efficacy of CH_4_-reducing feed additives due to CO_2_ prediction bias associated with energy utilization efficiency. We hypothesized that incorporating dry matter intake (DMI), along with metabolic body weight (MBW) and energy-corrected milk (ECM) as explanatory variables, would reduce this bias. The primary objective was to compare the performance of CO_2_ emission models with and without including DMI. The secondary objective was to assess the CO_2_-based method’s applicability for quantifying CH_4_-reducing effects, through a case study of 3-nitrooxypropanol (3-NOP).

**Methods:**

Prediction models for CO_2_ emissions were developed including DMI, MBW, and ECM as explanatory variables, based on 219 records obtained from previous experiments with Holstein cows using respiration chambers or headboxes. The model performance was evaluated using cross-validation. Bias associated with energy utilization efficiency was assessed. The applicability of the CO_2_-based method to quantify the CH_4_-reducing effect of 3-NOP was assessed using data obtained from the literature, including 10 studies with 22 treatment and control mean comparisons. The agreement between the observed and predicted CH_4_ reductions was assessed.

**Results:**

Combining DMI along with MBW and ECM improved the predictive performance of CO_2_ emissions. While the models without DMI showed bias associated with energy utilization efficiency, it was eliminated when DMI was incorporated. Applicability assessment demonstrated that the models without DMI systematically underestimated the CH_4_-reducing effect of 3-NOP. In contrast, the models with DMI showed smaller discrepancies between observed and predicted CH_4_ reductions.

**Conclusion:**

This study highlights the importance of incorporating DMI as an explanatory variable to achieve accurate and unbiased predictions of CO_2_ emissions. These findings would contribute to the appropriate application of the CO_2_-based method for evaluating the CH_4_-reducing effects of feed additives.

## INTRODUCTION

The use of methane (CH_4_)-reducing feed additives is one of the most effective strategies to mitigate enteric CH_4_ emissions from cattle [1,2]. The efficacy of these additives, such as 3-nitrooxypropanol (3-NOP), can be evaluated using direct gas flux measurement methods, including respiration chambers and the GreenFeed system [3]. However, these gas measurement methods can be challenging to implement in certain situations because of their installation and maintenance costs. The sniffer method, which involves sampling a portion of an animal’s exhaled breath, is a cost-effective technique for estimating CH_4_ emissions [4]. By combining the CH_4_:CO_2_ concentration ratio in the exhaled air with predicted CO_2_ emissions, individual CH_4_ emissions can be calculated [5,6]. Therefore, the accuracy and precision of CH_4_ emission estimates are highly dependent on the reliability of CO_2_ emission predictions.

Madsen et al [5] proposed a method for predicting CO_2_ emissions from dairy cows using heat production (HP) as an intermediate variable, where HP was estimated based on metabolic body weight (MBW), energy-corrected milk (ECM), and days in pregnancy. A fixed conversion factor was then applied to the estimated HP to derive CO_2_ emissions. This HP-based model assumes constant energy utilization efficiencies for maintenance and production. Additionally, it assumes a parallel relationship between HP and CO_2_ emissions, which, in practice, varies with the type of substrate being metabolized. Therefore, CO_2_ emissions predicted using the HP-based method exhibit bias due to variations in energy utilization efficiency and energy balance. Huhtanen et al [7] demonstrated that this method tends to overestimate CO_2_ emissions in efficient cows relative to inefficient cows, highlighting the challenges of applying CH_4_ prediction using the HP-based CO_2_ method in the context of genetic selection aimed at both reducing CH_4_ emissions and increasing productivity.

The issue of bias in using the HP-based CO_2_ prediction method may also be relevant when evaluating the efficacy of CH_4_-reducing feed additives. Specifically, the CH_4_-reducing potential of additives that simultaneously influence energy utilization efficiency may be evaluated with bias when CH_4_ emissions are quantified using the HP-based CO_2_ method. For example, if the amount of CH_4_ reduced by 3-NOP, which has already been demonstrated to significantly reduce CH_4_ emissions [8], is evaluated using the HP-based CO_2_ method, then the magnitude of the CH_4_ reduction may be underestimated. This underestimation arises because 3-NOP not only reduces CH_4_ emissions but also improves feed efficiency [9], thereby possibly influencing energy utilization efficiency, which is assumed to be constant in the HP-based method.

As MBW and ECM are energy consumption-related parameters, incorporating an energy intake-related parameter, such as dry matter intake (DMI), would account for variations in energy utilization efficiency and energy balance, thereby potentially correcting biases in predicted CO_2_ emissions associated with these factors. Although Kjeldsen et al [10] developed a CO_2_ emission prediction model that included DMI as an explanatory variable, it has not yet been demonstrated whether the inclusion of DMI effectively eliminates these biases. To determine the applicability of the CO_2_-based method for evaluating CH_4_-reducing feed additives, the influence of DMI on the CO_2_ prediction should be verified. We hypothesized that including DMI along with MBW and ECM as explanatory variables would eliminate the CO_2_ prediction biases, thereby enabling an unbiased assessment of the magnitude of CH_4_-reducing effects, even in the case of feed additives such as 3-NOP, which may influence energy utilization efficiency.

The primary objective of this study was to develop and evaluate CO_2_ emission prediction models with and without DMI as an explanatory variable. Model evaluation focused on the effect of incorporating DMI along with MBW and ECM on systematic biases associated with energy utilization efficiency and energy balance. The secondary objective was to assess the applicability of the CO_2_-based method for quantifying CH_4_-reducing effects, through a case study focusing on 3-NOP. Specifically, we examined how closely the CH_4_-reducing effect of 3-NOP, estimated using the CO_2_-based method, aligned with that evaluated using direct gas measurement methods.

## MATERIALS AND METHODS

This study used data from experiments previously conducted at the National Agriculture and Food Research Organization (NARO), as well as published data obtained through a literature search. Since no new animal experiments were performed, ethical approval from the Animal Care and Use Committee of NARO was not required.

### Prediction models for CO_2_ emissions

#### Dataset

This study utilized existing data collected from lactating Holstein cows at NARO research sites. From these datasets, records containing CO_2_ emissions, body weight (BW), milk yield and composition, DMI, gross energy intake (GEI), and retained energy were selected [11–22]. Retained energy was determined using the indirect calorimetry method, calculated as metabolizable energy intake minus the sum of HP and energy output in milk. Detailed information on each study included in the dataset, such as the experimental site, type of apparatus used for gas measurements (whole-body respiration chambers or headboxes), and main dietary ingredients, were described by Oikawa et al [23]. Using the formula proposed by Sjaunja et al [24], ECM (kg/d) was calculated as 0.25×milk yield (kg/d)+12.2×milk fat (kg/d)+7.7×milk protein (kg/d). Subsequently, the dataset was screened by performing a linear regression using CO_2_ emissions as the response variable, with MBW and ECM included as explanatory variables. Records with linear regression residuals exceeding three standard deviations were excluded as outliers. The resulting 219 records, including 211 from respiration chambers and 8 from headboxes, were used for model development. The descriptive statistics for the dataset are presented in Table 1.

#### Model development

Two modeling approaches were employed to develop CO_2_ emission prediction equations. The first directly predicted CO_2_ emissions using a linear mixed model (LMM). The second involved correction of the bias in CO_2_ emissions predicted by the HP-based method, expressed as: CO_2_ emissions = (bias-correction equation) × (HP-based CO_2_ emissions). This bias correction model was implemented using a generalized LMM (GLMM). The LMM and GLMM were fitted using the “lmer” and “glmer” functions, respectively, from the “lme4” package [25] in R software [26].

In the LMM approach, the response variable was CO_2_ emissions (g/d), and the explanatory variables included DMI (kg/d), MBW (kg^0.75^), and ECM (kg/d). The nested structure of the dataset was accounted for by including a random effect for the study group, defined by the year and site of the experiments conducted. Two models were developed using the LMM approach: one excluding DMI (Eq. i) and one including DMI (Eq. ii) as an explanatory variable.

In the GLMM approach, the HP-based CO_2_ emissions (g/d) were initially calculated based on MBW (kg^0.75^) and ECM (kg/d) using the following equation [5]:


(1) $$\begin{array}{l} HP-based\;CO_{2}\;emissions=\\ (24\times 180\times [5.6\times MBW+22\times ECM]/1,000)\times (44/22.4), \end{array}$$

where 24 is the number of hours in a day; 180 is liters of CO_2_ per 1,000 W of HP per hour; the term 5.6×*MBW*+22×*ECM* represents the estimated HP in units of 1 W; and 44/22.4 converts liters to grams. Although the equation proposed by Madsen et al [5] includes a variable for days in pregnancy, it was not considered in this study because of its small contribution and the lack of clear pregnancy data for some individuals in the dataset. The GLMM was fitted using the observed CO_2_ emissions (g/d) as the response variable and DMI (kg/d), MBW (kg^0.75^), and ECM (kg/d) as explanatory variables. The HP-based CO_2_ emissions were incorporated as an offset variable. The response variable was assumed to follow a gamma distribution. The model is expressed using a log-link function as follows:


(2) $$\begin{array}{l} \text{log}(CO_{2})=\beta_{0}+\beta_{1}\times DMI+\beta_{2}\times MBW+\beta_{3}\times ECM+\\ \text{log}(HP-based\;CO_{2}\;emissions)+study, \end{array}$$

where log(*CO_2_*) is the logarithm of the expected value of CO_2_ emission; *β*_0_ is the intercept; *β*_1_ to *β*_3_ are the regression coefficients; and *study* is the random effect of the study group. The model can be transformed as follows:


(3) $$\begin{array}{l} CO_{2}=\text{exp}(\beta_{0}+\beta_{1}\times DMI+\beta_{2}\times MBW+\beta_{3}\times ECM+study)\times \\ (HP-based\;CO_{2}\;emissions), \end{array}$$

This equation can be interpreted as follows: CO_2_ emissions = (bias-correction equation) × (HP-based CO_2_ emissions). Two models were developed using the GLMM approach: one excluding DMI (Eq. iii) and one including DMI (Eq. iv) as an explanatory variable.

#### Model performance

The model performance was evaluated using k-fold cross-validation, with each fold corresponding to a specific study group. Prediction accuracy was assessed using the root mean square error (RMSE). To facilitate interpretation, RMSE values are reported as a percentage of the mean observed values. The coefficient of determination (R^2^) was also calculated. To avoid overfitting, the R^2^ value of each model was compared with that of the reduced models generated by excluding individual variables from the full model. The model with the highest R^2^ value among the full and reduced models is reported in the Results. The concordance correlation coefficient (CCC) was calculated to assess both accuracy and precision [27]. The CCC evaluates how closely the regression line aligns with the identity line (y = x), with values closer to 1 indicating better model performance.

To evaluate bias in CO_2_ emissions, the residuals, defined as the difference between the observed and predicted CO_2_ emissions, were calculated. Bias associated with energy balance was investigated by plotting these residuals against the retained energy values, where values below zero represent a negative energy balance and values above zero represent a positive energy balance. The extent of bias was evaluated using the statistical significance of the slope of the linear regression between the residuals and retained energy values. Additionally, bias related to energy utilization efficiency was assessed. Given the large variation in feeding levels and energy balance among the cows in the dataset, the difference between the observed and expected ECM values was adopted as an indicator of energy utilization efficiency. The expected ECM was calculated using GEI (MJ/d), MBW (kg^0.75^), and retained energy (MJ/d) as follows [7]:


(4) $$\begin{array}{l} Expected\;ECM=\\ \beta_{0}+\beta_{1}\times GEI+\beta_{2}\times MBW+\beta_{3}\times RE_{p}+\beta_{4}\times RE_{n}+study, \end{array}$$

where *β*_0_ is the intercept (*β*_0_ = 4.64); *β*_1_ to *β*_4_ are the regression coefficients (*β*_1_ = 0.095, *β*_2_ = −0.084, *β*_3_ = −0.080, and *β*_4_ = −0.114); *RE_p_* is the retained energy value when positive; and *RE_n_* is the retained energy value when negative. If the observed ECM was higher than the expected ECM, it indicated greater milk production efficiency relative to animals with similar GEI, MBW, and energy balance levels.

### Applicability assessment of the CO_2_-based method: a case study

#### Data sources and extraction

Data for assessing the applicability of the CO_2_-based method to evaluate the CH_4_-reducing effect of 3-NOP were obtained through a literature search. Studies that used 3-NOP as a CH_4_-reducing feed additive and that reported CH_4_ and CO_2_ emissions were extracted from the de Ondarza et al [28] database. Consequently, six studies published until 2022 were obtained. Additionally, a literature search was conducted using Web of Science (https://www.webofscience.com/wos/woscc/basic-search) with the following title-based search criteria: (“NOP” OR “nitrooxypropanol” OR “Bovaer”) AND (“methane” OR “gas”) AND (“cows” OR “cattle”) NOT (“beef” OR “feedlot”) NOT (*in vitro*). The search was refined to include publications published from 2022 to 2025 and document types classified as articles, yielding 15 studies. Of a total of 21 studies obtained from the existing database and literature search, 11 studies were excluded for the following reasons: lack of CO_2_ emission data (n = 2); lack of BW data (n = 2); lack of control group (n = 1); nonlactating cow study (n = 1); grazing cow study where estimated DMI was reported (n = 1); including the breed other than Holstein (n = 1); meta-analysis (n = 1); manure study (n = 1); and study using data from previously published data (n = 1). The final dataset consisted of 10 studies, containing 22 comparisons between the treatment and control means [29–38]. The dataset included 17 control and 22 treatment means, with the discrepancy resulting from some treatment means sharing a common control. The descriptive statistics for the control and treatment means in the dataset are presented in Table 2. The gas measurement methods in the dataset included the use of respiration chambers (n = 2 studies) and the GreenFeed system (n = 8 studies).

#### Predicting CH_4_ emissions using the CO_2_-based method

To assess the reliability of the CH_4_-reducing effect estimated using the CO_2_-based method, the CH_4_ emissions in both the control and treatment groups for individual studies were calculated by multiplying the CH_4_:CO_2_ ratio by the predicted CO_2_ emissions. The CH_4_:CO_2_ ratios in the control and treatment groups were calculated by dividing the observed CH_4_ emissions by the observed CO_2_ emissions. The models developed in this study (Eqs. i–iv) and the HP-based model [5] were used to predict CO_2_ emissions. In addition, the CO_2_ prediction equations presented by Kjeldsen et al [10] were applied. They reported three CO_2_ prediction models covering different scenarios, based on the availability of DMI and BW. The highest data availability model (“best model”) included DMI, MBW, diet crude protein, breed, and parity as explanatory variables. The model without DMI (“on-farm model”) included ECM, MBW, milk fat, days in milk (DIM), breed, diet crude fat, and parity. The model without both DMI and BW (“reduced on-farm model”) included ECM, DIM, breed, parity, and diet crude fat.

For studies that did not report dietary chemical composition values, estimations were made based on the nutrient composition of feeds provided by the National Academies of Sciences and Medicine [39]. When only the overall BW value was reported, a common BW value was used for both the control and treatment groups. As this study utilized mean values from groups consisting of cows with mixed parities and could not determine the parity for each group, parity was fixed at second for all calculations.

#### Assessing the predicted CH_4_-reducing effect size

The CH_4_-reducing effect size estimated using the CO_2_-based method was compared with that evaluated using the direct gas measurement methods. The effect size was calculated as the mean difference (MD, g/d) of the CH_4_ emissions, defined as follows:


(5) $$\begin{array}{l} MD=Mean\;CH_{4}\;emissions\;in\;control\;group-\\ Mean\;CH_{4}\;emissions\;in\;treatment\;group \end{array}$$

This calculation was performed using both observed CH_4_ emissions (measured using respiration chambers or the GreenFeed system) and predicted CH_4_ emissions (derived from the CO_2_-based method). To assess the agreement between the observed and predicted CH_4_-reducing effect, the relative difference between the predicted and observed MD was calculated in percentage units as follows:


(6) $$\begin{array}{l} Relative\;difference\;in\;MD=([Predicted\;MD-\\ Observed\;MD]/Observed\;MD)\times 100 \end{array}$$

The relative difference in MD indicated how closely the CH_4_-reducing effect size calculated from the predicted CH_4_ emissions aligned with that derived from the observed CH_4_ emissions. Values near zero indicated good agreement, whereas positive or negative values indicated that the CO_2_-based prediction method overestimated or underestimated the true CH_4_-reducing effect, respectively.

To synthesize the relative differences in MD across individual studies, statistical analysis was performed using a random-effects model fitted with “lmer” function from the “lme4” package in R software. As the dataset contained multiple control and treatment comparisons within individual studies, the study was incorporated as a random effect. The model is expressed as follows:


(7) Relative difference in MD=Intercept+Study+Residual.

Intercept values closer to zero indicate an agreement between the CH_4_-reducing effect size estimated using the CO_2_-based method and that evaluated using direct gas measurement methods. To account for differences in the reliability of the MD across studies, weights were incorporated as the inverse of the sample size for each experiment.

## RESULTS AND DISCUSSION

### Prediction models for CO_2_ emissions

The CO_2_ emission prediction models developed in this study are listed in Table 3. Eqs. i, ii were developed using the LMM approach, while Eqs. iii, iv were developed using the GLMM approach, in the following form: CO_2_ emissions = (bias correction equation) × (HP-based CO_2_ emissions). All the regression coefficients in Eqs. i, ii were positive. In Eq. iii, the regression coefficient for MBW was positive, whereas that for ECM was negative. For Eq. iv, DMI, MBW, and ECM were initially included as explanatory variables. However, as the model excluding MBW had a higher R^2^ value than the full model, the model using DMI and ECM is presented. In this model, the regression coefficient for DMI was positive, while that for ECM was negative.

An example of using Eq. iv to calculate CO_2_ emissions for a cow with a DMI of 20 kg/d, BW of 700 kg, and ECM of 30 kg/d is as follows (Example 1):


(8) $$\begin{array}{l} {\text{CO}}_{2}\;\text{emissions }(\text{g}/\text{d})=\text{exp}(-0.0995+0.0300\times 20-0.0150\times \\ 30)\times (\text{HP-based }{\text{CO}}_{2}\;\text{emissions})=1.052\times 12,070. \end{array}$$

Another example using Eq. iv for a cow with a DMI of 20 kg/d, BW of 700 kg, and ECM of 35 kg/d (adding 5 kg/d from the previous example) is as follows (Example 2):


(9) $$\begin{array}{l} {\text{CO}}_{2}\;\text{emissions }(\text{g}/\text{d})=\text{exp}(-0.0995+0.0300\times 20-0.0150\times \\ 35)\times (\text{HP}-\text{based }{\text{CO}}_{2}\;\text{emissions})=0.976\times 13,004. \end{array}$$

As illustrated in these examples, Eq. iv adjusts the HP-based CO_2_ emissions upward (e.g., multiplied by 1.052 in Example 1) for cows with lower ECM levels relative to DMI and downward (e.g., multiplied by 0.976 in Example 2) for cows with higher ECM levels relative to DMI. High ECM levels relative to DMI typically occur when energy utilization efficiency is high or when energy balance is negative. This relationship indicates that Eq. iv adjusts the HP-based CO_2_ emissions downward for cows under conditions of high energy utilization efficiency or negative energy balance.

The predictive performances of the CO_2_ emission models are summarized in Table 3. The models that did not include DMI (Eqs. i, iii) had an R^2^ of 0.77 and a CCC of 0.76. The models that included DMI (Eqs. ii, iv) exhibited relatively high performance, with an R^2^ of 0.86 and a CCC of 0.85. These results are consistent with those reported by Kjeldsen et al [10], confirming that including DMI along with MBW and ECM clearly improves the predictive performance of CO_2_ emissions.

**Figure 1:**
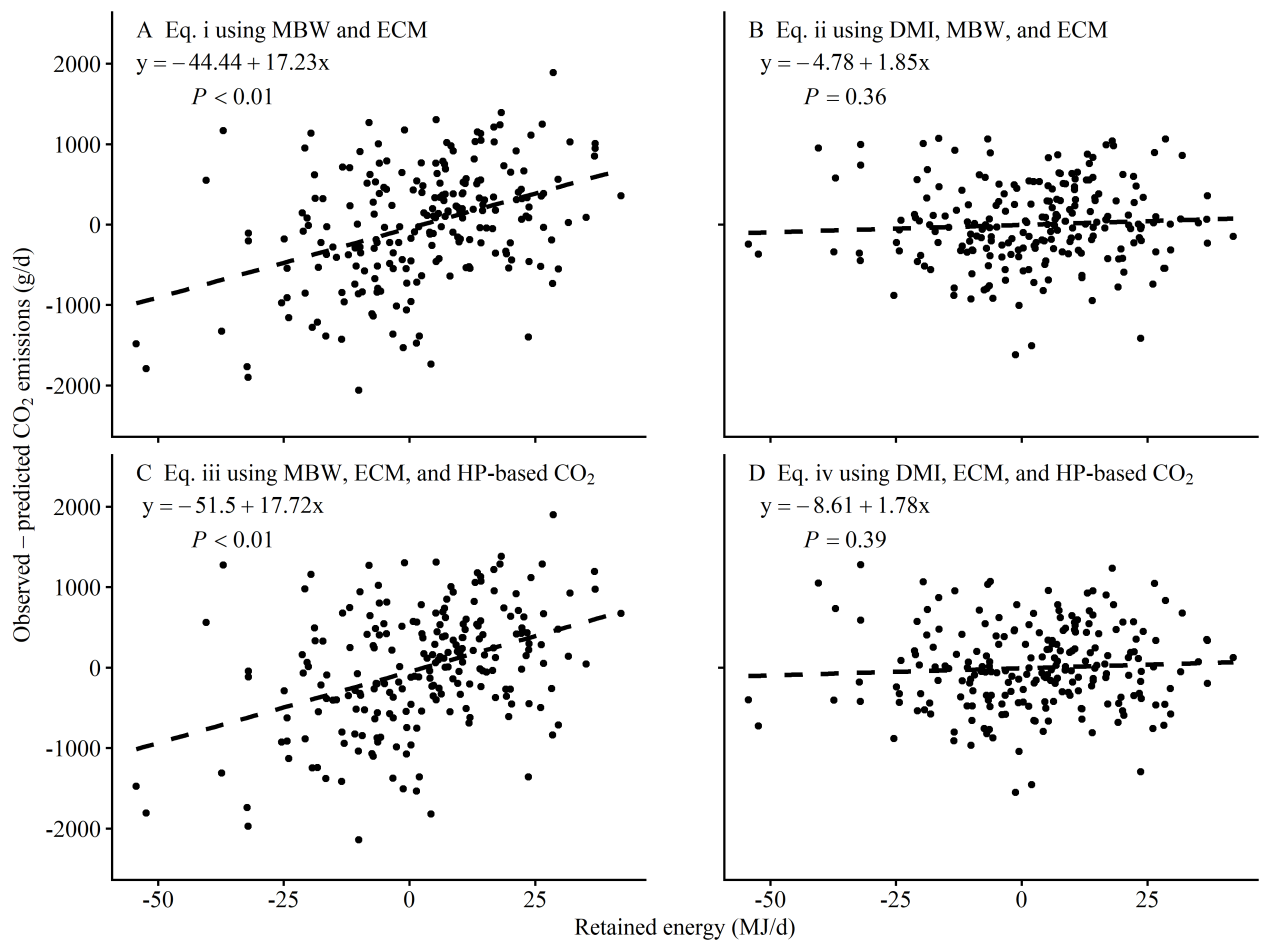
Bias in predicted CO_2_ emissions associated with retained energy. The dashed line indicates the regression line, with the p-value denoting the statistical significance of the slope. MBW, metabolic body weight; ECM, energy-corrected milk; DMI, dry matter intake; HP, heat production.

Bias in predicted CO_2_ emissions associated with retained energy is presented in Figure 1. When CO_2_ emissions were predicted without DMI, the regression slopes were significantly positive (p<0.01; Figures 1A, 1C), indicating a systematic overestimation of CO_2_ emissions in cows with a negative energy balance and an underestimation in cows with a positive energy balance. For instance, assuming a cow with a retained energy value of −25 MJ, Eq. i would overestimate CO_2_ emissions by 475.2 g/d (calculated as −44.44+17.23×–25; Figure 1A). These results align with physiological expectations, as fat mobilization occurs under negative energy balance, reducing actual CO_2_ production per unit of heat generated in the body [40]. In contrast, when DMI was included in the prediction models, the slopes of the regression were not significant (Figures 1B, 1D), suggesting that incorporating DMI in combination with MBW and ECM helped to eliminate the CO_2_ prediction bias related to energy balance. This elimination of the bias can be explained by the characteristics of the models that include DMI, in which CO_2_ emissions are predicted to be relatively low for cows under negative energy balance, as previously illustrated using examples based on Eq. iv.

**Figure 2:**
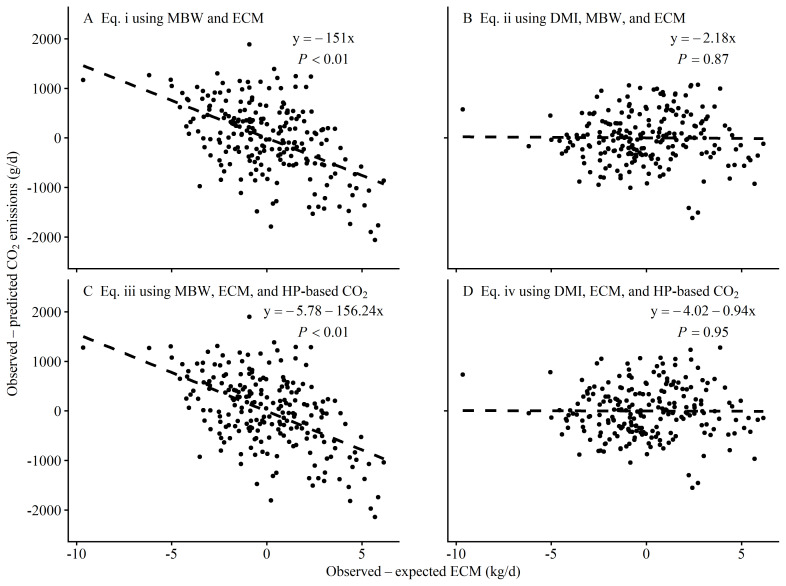
Bias in predicted CO_2_ emissions associated with energy utilization efficiency. The difference between the observed and expected energy-corrected milk (ECM) was used as an indicator of energy utilization efficiency. The dashed line indicates the regression line, with the p-value denoting the statistical significance of the slope. The intercept was not represented for (A) and (B), as its absolute value was less than 0.01. MBW, metabolic body weight; ECM, energy-corrected milk; DMI, dry matter intake; HP, heat production.

Bias in predicted CO_2_ emissions associated with energy utilization efficiency, expressed as the difference between the observed and expected ECM, is shown in Figure 2. The regression slopes were significantly negative in the models that did not include DMI (p<0.01; Figures 2A, 2C), indicating a systematic overestimation of CO_2_ emissions when energy utilization efficiency was high and an underestimation when it was low. For instance, assuming that a cow’s observed ECM was 5 kg higher than the expected ECM, Eq. i would overestimate CO_2_ emissions by 755 g/d (calculated as −151×5; Figure 2A). These results are consistent with the findings of Huhtanen et al [7] and seem reasonable, as cows with high milk production efficiency generate less heat during maintenance and production processes, and are therefore expected to produce less CO_2_ per MBW and ECM. In contrast, the slopes were not significant in the models that included DMI (Figures 2B, 2D), suggesting that incorporating DMI along with MBW and ECM effectively corrected the CO_2_ prediction bias related to energy utilization efficiency.

The results confirmed that the combination of MBW and ECM alone, both indicators of energy consumption, cannot account for the differences in CO_2_ emissions arising from variations in energy utilization efficiency and energy balance. Consistent with our hypothesis, incorporating DMI along with MBW and ECM improved the predictive performance of CO_2_ emissions and eliminated biases associated with energy utilization efficiency and energy balance.

### Applicability assessment of the CO_2_-based method: a case study

The relative differences between the CH_4_-reducing effect sizes estimated using the CO_2_-based method and those derived from the direct gas measurement methods are presented in Table 4. In this table, the models are listed in descending order based on the absolute value of the intercept, with values closer to zero indicating strong agreement between the observed and predicted CH_4_-reducing effect sizes. The models that did not include DMI underestimated the CH_4_-reducing effect, with underestimation rates of 11.56% for the HP-based model, 7.70% for the “reduced on-farm model”, 6.56% for Eq. i, 6.08% for Eq. iii, and 4.32% for the “on-farm model.” In contrast, the models that included DMI showed smaller discrepancies between the observed and predicted effect sizes, with relative differences of −3.53% for Eq. ii, −2.56% for Eq. iv, and −0.70% for the “best model.”

The results of the applicability assessment showed that the prediction models that did not include DMI systematically underestimated the CH_4_-reducing effect size of 3-NOP. This underestimation appears to be attributable to the difference in CO_2_ emissions per unit of ECM between the control and 3-NOP treatment groups. Based on the values presented in Table 2, CO_2_ emissions per unit of ECM were calculated as 387 g/kg (calculated as 13,702/35.4) for the control and 367 g/kg (calculated as 13,340/36.3) for the treatment, indicating that the use of 3-NOP reduced CO_2_ emissions per unit of ECM. Although the mechanism underlying this reduction is unclear, it may be related to improved energy utilization efficiency associated with 3-NOP supplementation. This interpretation is supported by the findings of a previous meta-analysis that reported an increase in ECM per unit of DMI using 3-NOP [9]. In any case, the results of this study indicate that unless other explanatory variables compensate for the difference in CO_2_ emissions per unit of ECM between the control and 3-NOP treatment groups, CO_2_ emissions in the treatment group would be overestimated compared to those in the control group, resulting in an underestimation of the CH_4_-reducing effect.

Importantly, the results demonstrated that the relative differences between the predicted and observed CH_4_-reducing effect sizes were minimized when DMI was included in the CO_2_ prediction models. This finding seems reasonable considering the results presented in Figure 2, which illustrate that incorporating DMI helps to eliminate the bias related to energy utilization efficiency. That is, DMI likely compensated for the difference in CO_2_ emissions per unit of ECM between the control and 3-NOP treatment groups, thereby correcting the underestimation of the CH_4_-reducing effect.

When interpreting the results of this study, it is important to acknowledge that the applicability of the CO_2_-based method was assessed through a case study on 3-NOP. We selected 3-NOP among various feed additives because it was demonstrated to reduce CH_4_ emissions and potentially influence energy utilization efficiency. These characteristics of 3-NOP were suitable for testing the hypothesis of this study. However, given that the assessment was based on a single case study on 3-NOP, one limitation is that the differences between the observed and predicted CH_4_-reducing effect sizes presented in Table 4 may not necessarily be the same for other feed additives. Future studies should assess the generalizability of the findings of this study using other types of CH_4_-reducing feed additives. Another limitation is that this study focused on issues related to predicted CO_2_ emissions and did not consider the measurement accuracy of the CH_4_:CO_2_ ratio, which is also an important factor for reliably predicting CH_4_ emissions. For evaluating the CH_4_-reducing effects of feed additives, ensuring the accuracy of the CH_4_:CO_2_ ratio measurements using the sniffer method is essential.

## CONCLUSION

In conclusion, as a first step toward applying the CO_2_-based method for evaluating the CH_4_-reducing effects of feed additives, this study demonstrates that the incorporation of DMI along with MBW and ECM as explanatory variables improves the predictive performance of CO_2_ emissions and eliminates the prediction biases associated with energy utilization efficiency and energy balance. Although further validation using CH_4_ reducing feed additives other than 3-NOP is required, the findings suggest that the CH_4_ emission prediction method based on CO_2_ emissions predicted using DMI could be a useful tool for evaluating the potential of CH_4_-reducing additives when direct gas measurement methods are not available.

## Figures and Tables

**Table 1:** Descriptive statistics of the dataset used for model development (n = 219)

Item	Unit	Mean	SD	Minimum	Median	Maximum
BW	kg	593	59	453	588	764
Milk yield	kg/d	25.3	6.6	6.1	25.4	41.1
Milk fat	%	3.7	0.5	2.4	3.7	5.3
Milk protein	%	3.1	0.3	2.1	3.0	3.9
ECM	kg/d	23.5	6.0	6.0	23.1	39.2
DMI	kg/d	16.8	3.4	6.1	17.3	23.7
Retained energy	MJ	3	17	−54	4	42
CO_2_ emissions	g/d	10,570	1,755	6,300	10,527	14,441

SD, standard deviation; BW, body weight; ECM, energy-corrected milk; DMI, dry matter intake.

**Table 2:** Descriptive statistics of the dataset^1)^ used for assessing the applicability of the CO_2_-based method for evaluating the methane (CH_4_)-reducing effect of 3-nitrooxypropanol

Item	Unit	Mean	SD	Minimum	Median	Maximum
BW, Con	kg	648	29	610	635	710
BW, Trt	kg	643	24	610	634	717
Milk yield, Con	kg/d	33.8	5.2	26.5	32.6	44.9
Milk yield, Trt	kg/d	35.0	6.6	25.8	35.4	45.8
Milk fat, Con	%	4.1	0.4	3.2	4.0	4.9
Milk fat, Trt	%	4.2	0.4	3.7	4.2	5.0
Milk protein, Con	%	3.5	0.3	3.0	3.6	4.1
Milk protein, Trt	%	3.5	0.4	3.0	3.4	4.1
ECM, Con	kg/d	35.4	3.2	30.9	34.9	42.7
ECM, Trt	kg/d	36.3	5.1	28.9	35.7	44.9
DMI, Con	kg/d	22.2	1.9	18.2	22.7	25.4
DMI, Trt	kg/d	21.8	2.5	17.1	21.1	26
CO_2_ emissions, Con	g/d	13,702	829	12,088	13,498	15,147
CO_2_ emissions, Trt	g/d	13,340	916	11,141	13,459	14,905
CH_4_ emissions, Con	g/d	391	54	272	406	487
CH_4_ emissions, Trt	g/d	280	44	216	268	361

1) The dataset included n = 17 control means and n = 22 treatment (3-nitooxypropanol) means, with the difference resulting from some treatment means sharing a common control.

SD, standard deviation; BW, body weight; Con, control; Trt, treatment; ECM, energy-corrected milk; DMI, dry matter intake.

**Table 3:** Models for predicting CO_2_ emissions

No.	Equations for CO_2_ emissions (g/d)	RMSE (%)	R^2^	CCC
i	−2,757+76.6×MBW+178×ECM	8.2	0.77	0.76
ii	−784+317×DMI+42.9×MBW+39.3×ECM	6.4	0.86	0.85
iii	exp(−0.206+0.00246×MBW−0.00154×ECM)×(HP-based CO_2_ emissions)	8.2	0.77	0.76
iv	exp(−0.0995+0.0300×DMI−0.0150×ECM)×(HP-based CO_2_ emissions)	6.4	0.86	0.85
Madsen et al [5]	HP-based CO_2_ emissions^1)^	9.3	0.75	0.70

1) HP-based CO_2_ emissions (g/d) = (24×180×[5.6×MBW+22×ECM]/1,000)×(44.01/22.4).

RMSE, root mean square error; R^2^, coefficient of determination; CCC, concordance correlation coefficient; MBW, metabolic body weight (kg^0.75^); ECM, energy-corrected milk (kg/d); DMI, dry matter intake (kg/d); HP, heat production.

**Table 4:** Relative difference between predicted and observed mean differences (MD) in methane (CH_4_) emissions

Model	Variables used in the CO_2_ emission prediction	Relative difference between predicted and observed MD (%)^1)^	

		Intercept	95% CI
Madsen et al [5]	MBW and ECM	−11.56	−14.8, −8.36
Reduced on-farm model [10]	ECM, DIM, breed, parity, and diet crude fat	−7.70	−11.5, −3.92
Eq. i in this study	MBW and ECM	−6.56	−10.2, −2.95
Eq. iii in this study	MBW and ECM	−6.08	−9.73, −2.44
On-farm model [10]	ECM, MBW, milk fat, DIM, breed, diet crude fat, and parity	−4.32	−8.70, 0.05
Eq. ii in this study	DMI, MBW, and ECM	−3.53	−8.25, 1.20
Eq. iv in this study	DMI, MBW, and ECM	−2.56	−8.47, 3.35
Best model [10]	DMI, MBW, diet crude protein, breed, and parity	−0.70	−6.22, 4.82

1) Calculated as: ([Predicted MD − Observed MD] / Observed MD)×100, where MD represents the control means minus treatment means in CH_4_ emissions. This value indicates how closely the CH_4_-reducing effect calculated from the predicted CH_4_ emissions aligns with that derived from the observed CH_4_ emissions. Values near zero indicated a good agreement, whereas positive or negative values indicated that the CO_2_-based prediction method overestimated or underestimated the CH_4_-reducing effect, respectively.

CI, confidence interval; MBW, metabolic body weight; ECM, energy-corrected milk; DIM, days in milk; DMI, dry matter intake.

## Data Availability

Upon reasonable request, the datasets of this study can be available from the corresponding author.
